# Acetaminophen-cysteine adducts during therapeutic dosing and following overdose

**DOI:** 10.1186/1471-230X-11-20

**Published:** 2011-03-14

**Authors:** Kennon J Heard, Jody L Green, Laura P James, Bryan S Judge, Liza Zolot, Sean Rhyee, Richard C Dart

**Affiliations:** 1 Rocky Mountain Poison and Drug Center, Denver Health, Denver CO, USA; 2 University of Colorado Department of Emergency Medicine, Aurora CO, USA; 3 Vanderbilt University School of Nursing, Nashville TN, USA; 4 Section of Pediatric Pharmacology and Toxicology, Arkansas Children's Hospital Little Rock, AR, USA; 5 Department of Emergency Medicine, Michigan State University College of Human Medicine, Spectrum Health-Toxicology Services, Grand Rapids, MI, USA; 6 Department of Emergency Medicine, Division of Medical Toxicology, University of Massachusetts Medical School, Worcester, MA, USA

## Abstract

**Background:**

Acetaminophen-cysteine adducts (APAP-CYS) are a specific biomarker of acetaminophen exposure. APAP-CYS concentrations have been described in the setting of acute overdose, and a concentration >1.1 nmol/ml has been suggested as a marker of hepatic injury from acetaminophen overdose in patients with an ALT >1000 IU/L. However, the concentrations of APAP-CYS during therapeutic dosing, in cases of acetaminophen toxicity from repeated dosing and in cases of hepatic injury from non-acetaminophen hepatotoxins have not been well characterized. The objective of this study is to describe APAP-CYS concentrations in these clinical settings as well as to further characterize the concentrations observed following acetaminophen overdose.

**Methods:**

Samples were collected during three clinical trials in which subjects received 4 g/day of acetaminophen and during an observational study of acetaminophen overdose patients. Trial 1 consisted of non-drinkers who received APAP for 10 days, Trial 2 consisted of moderate drinkers dosed for 10 days and Trial 3 included subjects who chronically abuse alcohol dosed for 5 days. Patients in the observational study were categorized by type of acetaminophen exposure (single or repeated). Serum APAP-CYS was measured using high pressure liquid chromatography with electrochemical detection.

**Results:**

Trial 1 included 144 samples from 24 subjects; Trial 2 included 182 samples from 91 subjects and Trial 3 included 200 samples from 40 subjects. In addition, we collected samples from 19 subjects with acute acetaminophen ingestion, 7 subjects with repeated acetaminophen exposure and 4 subjects who ingested another hepatotoxin. The mean (SD) peak APAP-CYS concentrations for the Trials were: Trial 1- 0.4 (0.20) nmol/ml, Trial 2- 0.1 (0.09) nmol/ml and Trial 3- 0.3 (0.12) nmol/ml. APAP-CYS concentrations varied substantially among the patients with acetaminophen toxicity (0.10 to 27.3 nmol/ml). No subject had detectable APAP-CYS following exposure to a non-acetaminophen hepatotoxin.

**Conclusions:**

Lower concentrations of APAP-CYS are detectable after exposure to therapeutic doses of acetaminophen and higher concentrations are detected after acute acetaminophen overdose and in patients with acetaminophen toxicity following repeated exposure.

## Background

Acetaminophen toxicity is a major cause of acute liver failure in the United States. While many cases have a clear history of acetaminophen exposure, there are a substantial number of cases in which the cause of liver injury is not clear. As serum acetaminophen concentrations may be undetectable by the time the patient has acute liver failure, there is a need for a biomarker with a longer detection time.

The metabolism of acetaminophen by CYP2E1 forms *N*-acetyl-*p*-benzoquinone imine (NAPQI), a reactive metabolite that binds to cysteine residues in cellular proteins and forms acetaminophen protein adducts, herein referred to acetaminophen-cysteine (APAP-CYS)[1]. Previous experimental work has shown that APAP-CYS is formed within hepatocytes and released into the circulation following cell necrosis in the setting of acute acetaminophen toxicity[2,3]. This product is detectable in serum for several days following acute overdose, and it has been proposed that detection of APAP-CYS is diagnostic of acetaminophen toxicity[4]. The majority of the studies measuring APAP-CYS have included patients with known or suspected acute overdose of acetaminophen[4-7].

While studies of patients with acetaminophen-induced liver injury can determine the sensitivity of the assay, the performance of the assay cannot be studied solely in this population. For a diagnostic study to be valid, the assay should be evaluated in patients who have similar diseases to the diagnosis of interest. Therefore, in this study, the assay was tested in other patient groups including patients with liver injury from other causes, patients taking therapeutic doses of acetaminophen and patients with liver injury following repeated ingestion of supra-therapeutic doses of acetaminophen. The objective of this study is to describe the serum concentrations of APAP-CYS measured under five conditions: 1) during therapeutic dosing, 2) in patients with suspected acetaminophen toxicity from repeated overdosing, 3) following acute acetaminophen overdose with early acetylcysteine treatment, 4) following acute acetaminophen overdose with delayed acetylcysteine treatment and 5) following ingestion of non-acetaminophen hepatotoxins.

## Methods

This report describes the analysis of samples collected in two settings: 1) during three clinical trials that included an acetaminophen treatment arm and 2) an observational study of patients with an overdose of acetaminophen or another hepatotoxin. The three clinical trials was separately reviewed and approved by the Colorado Multiple Institutional Review Board. Trial 3 was also approved by the Center For Addition and Mental Health Research Ethics Board. The observational study was approved by the Colorado Multiple Institutional Review Board and the Spectrum Health Research and Human Rights Committee. All subjects provided informed consent for inclusion in the trials and in the observational study.

### Study methods for clinical trials

The populations, duration of dosing and days of sampling for all trials are shown in Table 1. The clinical trials were performed to evaluate the effect of acetaminophen on serum transaminase activity. In all clinical trials, healthy adult subjects older than 21 years were administered the maximum daily dose of acetaminophen (4 g/day) or placebo (only Trials 2 and 3 had a placebo arm). The study subjects were non-drinkers (Trial 1-Registration number NCT 00616018)[8], moderate drinkers (Trial 2 Registration number NCT 004000621)[9] and subjects who chronically abuse alcohol (Trial 3- Registration number NCT 00427206)[10]. For Trial 2, moderate drinking was defined as 1-3 alcoholic beverages per day. For Trial 3, subjects who chronically abuse alcohol were identified at an inpatient detoxification facility; the average duration of their current drinking binge was longer than 1 month. Subjects in Trials 1 and 3 could not consume ethanol during the study; subjects in Trial 2 were instructed to continue their usual drinking pattern during the study (an average of 1-3 drinks/day). The first acetaminophen or placebo dose was administered on study day 1 in all trials.

**Table 1:** Characteristics and peak recorded serum APAP-CYS concentrations for subjects administered 4 g/day of acetaminophen during three clinical trials.

	Study		
	**Trial 1**	**Trial 2**	**Trial 3**

Subjects	Non-drinkers	Moderate drinkers	Alcoholics

Duration of dosing	10 d	10 d	5 d

Days of sampling	0, 4, 7, 9, 11,14	0,11	0, 2, 4, 6,7

n	24	91 APAP 47 placebo	40 APAP/ 7 placebo

Median age (range) years	34.5 (23 to 63)	30 (21 to 64)	46 (33 to 66)

Male	7 (29%)	47 (52%)	39 (98%)

ALT			

Baseline ALT Median (Range) IU/L	24 (14 to 45)	19 (11 to 49)	29 (10 to 178)

Peak ALT Median (Range) IU/L	45 (19 to 136)	25 (14 to 128)	47.5 (8 to 238)

ALT >ULN during study n (%)	14 (58%)	17 (19%)	24 (60%)

Peak Serum APAP-CYS			

Mean (SD) nmol/L	0.4 (0.20)	0.1 (0.09)*	0.3 (0.12)

Range nmol/L	0.1 to 1.0	0.0 to 0.5	0.1 to 0.8

Trial 1 included non-drinkers treated for 10 days, Trial 2 included moderate drinkers treated for 10 days and Trial 3 included alcoholic subjects treated for five days. APAP-CYS-Acetaminophen-cysteine adducts, ALT-alanine aminotransferase, ULN-upper limits of normal for the test. SD-standard deviation.

*p < 0.001 vs Trial 1 and 3

### Study methods for observational study

In the observational study, patients were enrolled by two medical toxicology services evaluating patients for known or suspected acetaminophen overdose. The following patients were eligible for enrollment: 1) patients following acute acetaminophen ingestion (defined as a single ingestion or multiple ingestions occurring within an 8 hour period), 2) patients with repeated acetaminophen ingestions (defined as multiple ingestions over a period of greater than 8 hours); 3) patients that may have ingested a product that may produce hepatotoxicity (other than acetaminophen). A trained investigator collected a structured data set for each patient subject including demographics, time course of ingestion, co-ingestions (including ethanol), medical history (including history of alcohol abuse and liver disease) and clinical course (including time of acetylcysteine treatment relative to the overdose, laboratory variables and outcome). Samples for APAP-CYS quantification were collected during routine blood draws, and therefore sample collection was not systematically timed. In this report we refer to the highest recorded determination as the peak value, but we recognize that our non-systematic sampling scheme may miss the true peak values.

The patients in the overdose cohort were stratified into the following four groups: 1) Acute acetaminophen ingestion who received acetylcysteine within 8 hours (acute early treatment), 2) acute acetaminophen ingestion who received acetylcysteine after 8 hours (acute late treatment), 3) repeated acetaminophen ingestion and 4) ingestion of a hepatotoxin without acetaminophen.

### Sample Preparation, APAP-CYS Protein Adduct Assay

Serum samples were frozen at each institution and stored at -80°C until analysis. Samples were analyzed using a previously described method[6]. The samples were processed with gel filtration, protein digestion and the resulting APAP-CYS concentration was determined by high pressure liquid chromatography using electrochemical detection. The limit of detection of the assay is 0.03 uM (micromolar) based on the lowest quality control concentration measured with a CV of within 15%. The final concentrations are reported as nmol of APAP-CYS/ml of serum to be consistent with previous publications.

### Analysis plan

For both the trials and the observational study, categorical variables are described as percentages with 95% exact binomial confidence intervals. Continuous variables that were non-skewed are reported using means with standard deviations while continuous variables with skewed distributions are described using medians with inter-quartile ranges. The mean peak adduct concentrations for the three therapeutic trials were compared using ANOVA with a protected Fisher's Least Significant Difference to adjust for multiple comparisons in post hoc tests. An overall, 2-sided p-value <0.05 was considered significant. The relationship of peak APAP-CYS concentrations to peak ALT for each trial was evaluated with Spearman's rho. If the 95% confidence intervals for rho included zero, we considered there to be no significant relationship between peak recorded ALT and peak recorded APAP-CYS. Calculations were performed using SAS version 9.2 (Carey, NC) and correlations were performed using Prism 5.0 (GraphPad Software, San Diego CA).

## Results

### Clinical trials

There were 144 samples from 24 subjects in Trial 1, 276 samples from 91 APAP and 47 placebo subjects in Trial 2, and 200 samples from 40 APAP and 7 placebo subjects in Trial 3. The demographics and peak recorded APAP-CYS concentrations in trial subjects are shown in Table 1.

At baseline, no subjects in Trial 1, 6 subjects in Trial 2 (2 acetaminophen, 4 placebo) and 5 subjects (1 placebo, 4 APAP) in Trial 3 had detectable APAP-CYS concentrations. Of these 11 APAP-CYS baseline positive subjects, 4 reported acetaminophen use in the 4 days preceding the study and had detectable APAP serum concentrations at baseline, 3 reported use in the 4 days preceding the study but did not have a detectable serum APAP concentration at baseline, 1 subject denied acetaminophen use, but had a detectable APAP concentration at baseline and 3 subjects denied acetaminophen ingestion and had no detectable acetaminophen at baseline.

**Figure 1:**
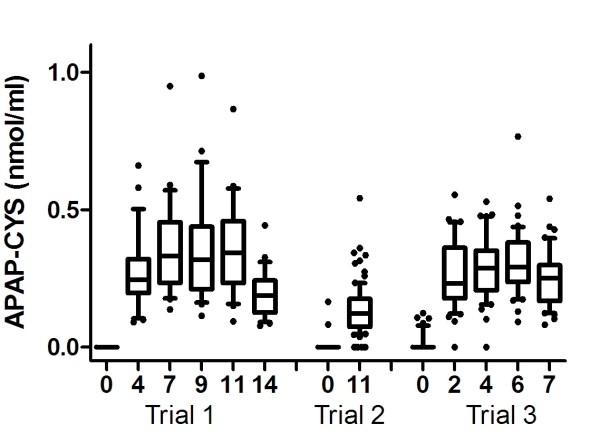
**Serum APAP-CYS concentrations for subjects in three trials**. Subjects received 4 g/day of acetaminophen for 10 days (Trials 1 and 2) or 5 days (trial 3) starting at day 1. Bars represent the 10-90^th ^percentile for each study and values outside that range are shown as '•'.

All acetaminophen-treated subjects (n = 115) in the Trials 1 and 3 had detectable APAP-CYS in one or more samples. Eighty five of 91 APAP-treated subjects in Trial 2 had detectable serum concentrations of APAP-CYS on day 11 of the trial. Thus, overall, APAP-CYS was detected in the vast majority of samples obtained from the APAP-treated arms of the 3 trials. The mean peak recorded adduct concentrations in Trial 2 were lower than concentrations measured in Trials 1 and 3 (p < 0.001, values shown in Table 1). However, there was substantial overlap of individual subject values between the trials and these differences are unlikely to be of clinical significance (Figure 1).

Two placebo treated subjects in Trial 2 and 1 placebo treated subject in Trial 3 had detectable APAP-CYS at the end of the study. All of these subjects had detectable APAP-CYS concentrations (0.03, 0.16, 0.17 nmol/ml) at baseline and the final concentrations (0.004, 0.06, 0.09 nmol/ml respectively) were lower than the baseline concentrations.

Mean APAP-CYS concentrations tended to increase during the dosing period of each study (Figure 1). In Trial 1, samples were collected 1 and 4 days after the final acetaminophen dose and in Trial 3 samples were collected 1 and 3 days after the final acetaminophen dose. In both of these trials, the APAP-CYS concentrations 1 day after dosing was stopped were similar to the concentrations measured during dosing, and samples obtained 3 or 4 days after stopping acetaminophen were lower than those observed on dosing (Figure 1).

**Figure 2:**
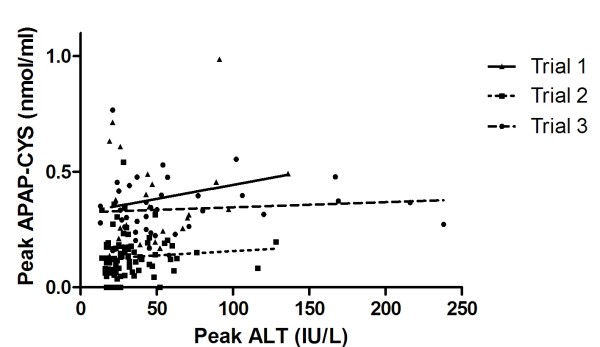
**Peak APAP-CYS concentration by peak ALT during three trials where subjects were administered 4 g/day of acetaminophen**.

Peak recorded APAP-CYS concentrations and peak recorded ALT for all subjects receiving therapeutic doses are shown in Figure 2. Please note that peak recorded values of APAP-CYS and ALT did not necessarily occur at the same time point so the values reported in Figure 2. The Spearman's rho correlation (with 95% CI) of the relationship between ALT and APAP-CYS for all trials was not significant: Trial 1 0.40 (-0.37 to 0.44); Trial 2 0.23 (-0.02 to 0.41) and Trial 3 0.14 (-0.18 to 0.43).

### Observational study

**Figure 3:**
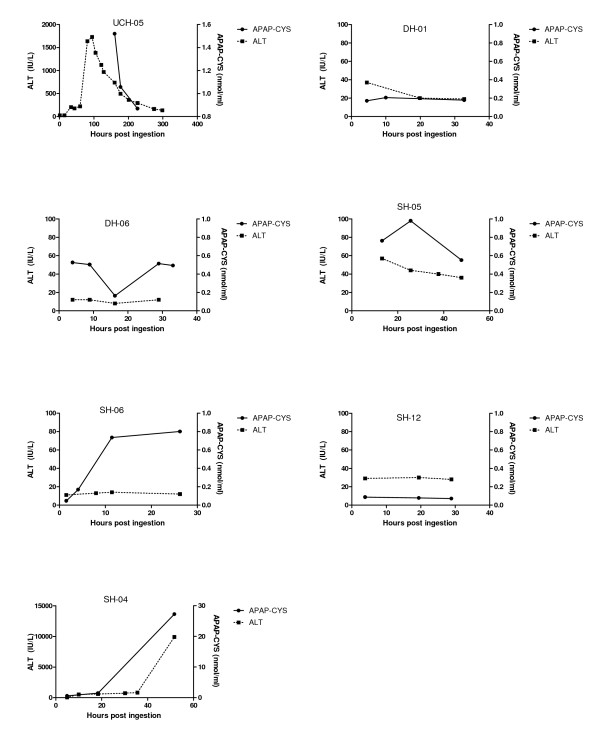
**Serum APAP-CYS concentrations and ALT activity for seven patients who received acetylcysteine treatment within 8 hours of their acute acetaminophen overdose**. Clinical characteristics of the cases are shown in Table 2. Note that the scale of the axes are different for subject SH-04 to reflect in the range of values for this subject.

**Figure 4:**
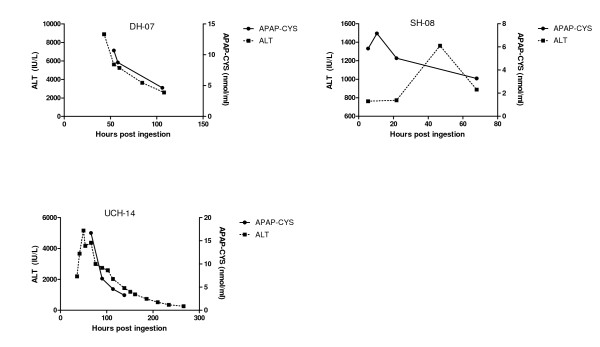
**Serum APAP-CYS concentrations and ALT activity for three patients who received acetylcysteine treatment more than 8 hours after their acute acetaminophen overdose**. Clinical characteristics of the cases are shown in Table 2. Note that the scale of the axes are not the same for all subjects to reflect the differences in the range of values.

**Figure 5:**
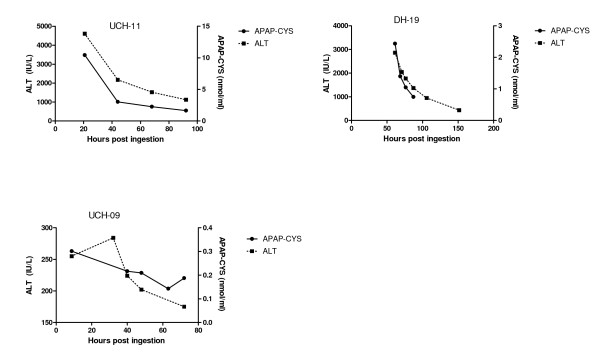
**Serum APAP-CYS concentrations and ALT activity for three patients who were treated with acetylcysteine for hepatic injury following repeated supratherapeutic ingestion of acetaminophen**. Clinical characteristics of the cases are shown in Table 2. Note that the scale of the axes are not the same for all subjects to reflect the differences in the range of values.

Thirty five patients were enrolled in the observational study; this included 31 acetaminophen overdoses and 4 subjects who had ingested another hepatotoxin. No serum samples were obtained for APAP-CYS analysis in 4 patients (all acetaminophen), so these patients were excluded. Note that the subject numbers shown in the tables and figures are non-consecutive because packets were pre-numbered; missing numbers reflect unused packets rather than exclude subjects. Among the remaining patients, the types of acetaminophen ingestion were: 12 acute early treatments (within 8 hours of exposure), 7 acute late treatments (more than 8 hours post exposure), 7 repeated ingestions and 1 unknown. This analysis included a total of 82 samples for APAP-CYS measurement. The number of samples for each patient ranged from 1 to 5. All of the patients who reported ingesting acetaminophen had detectable serum APAP-CYS and none of the 4 patients who reported ingesting hepatotoxins without acetaminophen had APAP-CYS detected (Table 2). Figures 3, 4, and 5 show the time course of serum APAP-CYS concentrations and ALT activity for selected patients in the acetaminophen overdose group who had three or more measurements. The figures are segregated by time to treatment with NAC (early, Figure 3), >8 hour (Figure 4), and by patients with supratherapeutic acetaminophen ingestions (Figure 5).

**Table 2:** Demographics and clinical characteristics of patients enrolled following acute overdose, repeated overdose or after ingestion of a non-acetaminophen hepatotoxin.

Patient number	Age/ Sex	Peak ALT IU/L	Case description
			**Acute acetaminophen treated within 8 hours of ingestion**

D-01	26 F	37	40 unknown strength tabs. 4.5 hr [APAP] 230 mcg/ml. 6.5 hrs to NAC.

D-02	20 F	18	20 g. 2.5 hr [APAP] 262 mcg/ml. 5 hr to NAC.

D-04	35 F	26	10-13 g. 6 hr [APAP] 177 mcg/ml. 7 hr to NAC.

D-06	20 F	17	Approx 30 tabs unknown strength. 4 hr [APAP] 319 mcg/ml. 6 hr to NAC.

SH-03	55 F	16	10.4 g. 4 hr [APAP] 184 mcg/ml. 6 hr to NAC.

SH-04	22 M	9903	25 g. 5 hr [APAP] 221 mcg/ml. 7 hr to NAC.

SH-05	38 F	57	39 g. 13 hr [APAP] 296 mcg/ml. 3.5 hr to NAC.

SH-06	16 F	14	"3 handfuls" of unknown product. 4 hr [APAP] 293 mcg/ml. 5.5 Hr to NAC.

SH-07	24 M	27	13-16.5 g. 6 hr [APAP] 235 mcg/ml. 8 hr to NAC.

SH-12	42 M	30	Unknown amount. 4 hr [APAP] 215 mcg/ml. 7 hr to NAC.

UCH-05	27 F	1729	48 gm. 1 hr [APAP] 557 mcg/ml. 5.5 hr to NAC.

UCH-06	51 F	22	19.5-53 g. 3 hr [APAP] 467 mcg/ml. 5 hr to NAC.

			**Acute acetaminophen treated more than 8 hours post ingestion**

D-05	25 F	18	Unknown amount. 8 hr [APAP] 373 mcg/ml. 9 hr to NAC.

D-07	38 M	8880	7 g. 41 hr [APAP] 9 mcg/ml. 49 hr to NAC. Hep C.

D-08	37 F	1999	5 g × 2 8 hrs apart. 12 hr (from first ingestion) [APAP] 121 mcg/ml. 13 hr to NAC.

SH-08	37 F	1362	13-16 g. 10 hr [APAP] 89 mcg/ml. Time to NAC 15 hr.

UCH-01	52 F	457	15-25 g. 24 hr [APAP] 10 mcg/ml. Time to NAC 26 hr.

UCH-07	40 F	2083	Unknown amt. 27 hr [APAP] 336 mcg/ml. Time to NAC 28 hr.

UCH-14	41 F	4372	30 g. 36 hr [APAP] 5.6 mcg/ml. Time to NAC 36 hr.

			**Repeated acetaminophen ingestion**

D-00	27 M	741	Approximately 10 g/day for 4 d for dental pain. Presenting [APAP] 0 mcg/ml.

D-18	17 M	229	Approximately 4 g/day for 2 d then 8 g/day for 1 day for prevertebral abscess. Presenting [APAP] 20 mcg/ml.

D-19	44 F	2862	4-6 g/day for 4 days then 25-50 g OD. 60 hr [APAP] 0 mcg/ml. NAC 14.5 hr post OD.

UCH-03	28 F	3189	2-3 tabs unknown str every 2-4 hrs for 7 d. Presenting [APAP] 16 mcg/ml.

UCH-08	29 F	2099	Approx 12 gm over 14 hrs. Presenting [APAP] 0 mcg/ml.

UCH-09	38 F	284	21 g over 5 d. Presenting [APAP] 0 mcg/ml. CMV hepatitis on liver biopsy.

UCH-11	24 M	7979	5-7.5 g/day for 4 d. presenting [APAP] 0 mcg/ml.

			**Acetaminophen ingestion-not able to determine time course**

UCH-15	29 F	131	Unknown amt over several hours. Presenting [APAP] 36 mcg/ml. Polypharmacy OD.

			**Ingestion of other hepatotoxins**

D-16	12 M	2346	Isoniazd-induced liver injury during therapeutic use.

SH-01	47 M	174	1500 mg of tramadol. Alcoholic

SH-02	24 M	18	9.5 g of valproic acid.

SH-09	18 F	18	8 g phenytoin.

[APAP]- serum acetaminophen concentration, NAC- acetylcysteine, ALT- alanine aminotransferase.

**Figure 6:**
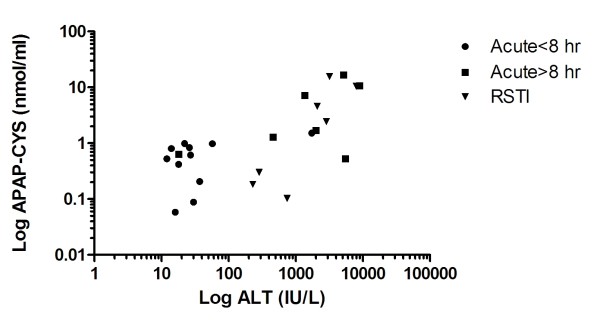
**Peak APAP-CYS concentrations plotted by peak ALT for patients treated within 8 hours of acute overdose, for patients treated more than 8 hours after acute overdose and patients treated for repeated supratherapeutic ingestion (RSTI)**.

As the values for both ALT and APAP-CYS were highly skewed in the overdose and toxicity patients, the values were log transformed for analysis. A plot of the log of adduct concentrations versus log of ALT is shown in Figures 6. Please note that peak recorded values of APAP-CYS and ALT did not necessarily occur at the same time point so the values reported in Figure 6. There was a modest Spearman correlation between the peak recorded adducts and peak recorded ALT (rho = 0.68, 95% CI 0.36 to 0.85).

## Discussion

Our study results demonstrate that low concentrations of APAP-CYS are detectable in serum following therapeutic dosing with acetaminophen in the vast majority of subjects. The peak recorded APAP-CYS concentrations are higher in non-drinkers and abstaining subjects who chronically abuse alcohol than in moderate drinkers, but the differences were small. The APAP-CYS concentrations following acetaminophen overdose varied widely. Serum APAP-CYS concentrations in patients with hepatic injury following acetaminophen overdose were generally much higher than those observed during therapeutic dosing. However, three overdose subjects (Subjects SH-05, SH-06, DH-06 in Figure 5) with normal serum ALT activity or minimal ALT elevation (due to early treatment with N-acetylcysteine or late sampling during resolution of injury) had APAP-CYS concentrations that were of the same order of magnitude as those observed with therapeutic dosing.

A previous report showed measureable APAP-CYS concentrations with therapeutic dosing[11]. Based on earlier published data in animal models, and early in the development of this assay, APAP-CYS formation was thought to occur only after acetaminophen overdose with hepatic injury[12,13]. However, as the sensitivity of the assay has improved[6,14], it is clear that that APAP-CYS formation occurs in the setting of low dose exposure associated without ALT activity[11].

An unexpected finding was that APAP-CYS concentrations were lower at study day 11 in Trial 2 than the end of study samples collected for the subjects in Trial 1 (study day 11) and Trial 3 (study day 7). No adducts were detected in six of the 91 APAP dosed patients on study day 11 in Trial 2. Slight differences in the sampling scheme among these three studies make direct comparisons difficult. It is possible that these differences are due to variations in sample timing, handling or processing or non-compliance with study medication. However, overall compliance with dosing in Trial 2 was similar to the compliance reported for Trial 1 (<1% of doses missed for both studies) and the six Trial 2 subjects with undetectable adduct concentrations did not report any missed doses. An alternative hypothesis is that ethanol exposure may have altered adduct formation. As ethanol is a known inhibitor of CYP 2E1, the enzyme primarily responsible for the formation of NAPQI with therapeutic doses of acetaminophen, it is possible that ethanol blocked NAPQI formation in the group allowed to consume ethanol during the study while the other two groups were not allowed to drink and thus had more metabolism of acetaminophen via CYP 2E1 to NAPQI This mechanism could be further investigated in future large prospective study that compared different ethanol exposures concurrent with APAP exposure and a single sampling scheme.

As no patients who ingested non-acetaminophen hepatotoxins had detectable serum APAP-CYS, our findings support prior publications suggesting that the serum APAP-CYS is specific for APAP exposure in overdose patients[4,12]. Our findings are limited as we only had 4 patients non-acetaminophen patients. In addition, most patients who received early treatment with NAC (Figure 3), had lower adduct concentrations than those that had late treatment with NAC (Figure 4). An exception to this generalization is patient SH-04 (Figure 3). This patient had a low ALT and APAP-CYS concentration at presentation but went on to develop a marked ALT elevation and high APAP-CYS concentration consistent with acetaminophen-induced liver injury. The placebo subjects who had APAP-CYS detected at the end of treatment during the therapeutic trials had measurable concentrations prior to starting the trial and the concentration decreased during the trial. We believe this finding to be consistent with acetaminophen ingestion prior to the trial.

A serum APAP-CYS concentration greater than 1.1 nmol/ml has a sensitivity of 97% for identification of patients with hepatotoxicity (defined as an ALT values of >1000 IU/L) when applied to a cohort of patients with acetaminophen overdose[6]. While the mean concentrations in our three therapeutic trials were well below this threshold, one patient in Trial 1 had a peak recorded APAP-CYS concentration of 1.0 nmol/ml and another patient in Trial 3 had a concentration of 0.8 nmol/ml. Further studies are required to determine if the high sensitivity of this assay threshold is maintained when applied to populations other than acetaminophen overdoses (i.e. therapeutic dosing).

During therapeutic trials, APAP-CYS concentrations increased during the dosing period. It is unknown whether a plateau occurs at some point or whether concentrations continue to rise as long as acetaminophen is consumed. In overdose cases, APAP-CYS concentrations generally decreased following the peak in late presenters and patients with repeated ingestions; the rate of decline closely follows that of ALT and AST. This is consistent with previous reports[6]. The concentrations showed a less consistent decrease in the acute presentations who were treated early.

We found no relationship between APAP-CYS concentrations and serum ALT activity (a commonly used marker of liver injury) in the three trials of therapeutic exposure. One interpretation of this lack of correlation is that low-level adduct formation is not a marker of liver injury. However, it is also possible that there is low level liver injury that is not reflected by changes in transaminases. At this time we cannot make conclusions regarding the lack of correlation between APAP-CYS and ALT. The correlation between APAP-CYS and ALT observed in the overdose cohort was statistically significant and similar to other reports[6,14]. For example, a correlation of 0.84[14] and 0.73[6] for ALT were previously reported in children and adults, respectively, with APAP overdose. Importantly, the highest concentrations of APAP adducts measured in patients were generally seen in those with severe cases of APAP overdose who did not receive timely treatment with NAC.

## Conclusion

Our findings support previous studies suggesting APAP-CYS is a specific marker of acetaminophen exposure, and that adduct concentrations vary according to the degree of exposure. Higher concentrations are associated with liver injury and APAP-CYS was detectable in acetaminophen overdose patients. Currently, an absolute adduct level exceeding 1.1 nmol/mL appears consistent with acetaminophen toxicity while therapeutic dosing generally produces concentrations below 0.5 nmol/mL. Clinical history, including the magnitude of the exposure, the timing of acetylcysteine administration, and laboratory markers of liver injury should also be taken into account when interpreting adduct concentrations in the lower range. A recent publication has described the use of APAP-CYS to identify occult acetaminophen poisoning among patenits with liver failure of unclear etiology[15]. It is likely that APAP-CYS will be a useful diagnostic test in cases in which the history and standard laboratory testing are not sufficient to establish acetaminophen as a cause of liver injury

There are other potential applications that should be investigated in future studies. We had one patient (D = 05) who was stratified as high risk (using the nomogram) but who had low adduct concentrations and who did not develop liver injury. While this is a single case, it suggests that a low APAP-CYS in high risk patient may be a marker that could be used to identify patients who do not need prolonged therapy. In our small cohort, we found that APAP-CYS concentrations were elevated in all patients who developed hepatoxicity (ALT >1000 IU/L) following repeated ingestions. This suggests that APAP-CYS may be useful for risk stratification following repeated overdose.

## Competing interests

This study was funded by an investigator-initiated research grant from McNeil Consumer Healthcare to Denver Health. The sponsor had no role in the design, performance, and analysis. The sponsor reviewed the paper for prior to publication, but the authors retained final responsibility for the manuscript. Dr. James has a patent for technology relating to the measurement of acetaminophen protein adducts.

## Authors' contributions

KH, JG, RCD designed the study and secured funding. KH, JG, RCD, BJ, SR collected the data. LJ performed the APAP-CYS determinations. KH and LZ performed the statistical analysis. KH, SR and JG drafted the manuscript and all authors contributed substantially to the revisions and reviewed the final manuscript.

## Pre-publication history

The pre-publication history for this paper can be accessed here:

http://www.biomedcentral.com/1471-230X/11/20/prepub
